# Exportin-1-Dependent Nuclear Export of DEAD-box Helicase DDX3X is Central to its Role in Antiviral Immunity

**DOI:** 10.3390/cells8101181

**Published:** 2019-09-30

**Authors:** Steven M. Heaton, Sarah C. Atkinson, Melissa N. Sweeney, Sundy N. Y. Yang, David A. Jans, Natalie A. Borg

**Affiliations:** 1 Infection & Immunity Program, Monash Biomedicine Discovery Institute and Department of Biochemistry and Molecular Biology, Monash University, Clayton, VIC 3800, Australia; steven.heaton@monash.edu (S.M.H.); sarah.atkinson@monash.edu (S.C.A.); melissa.sweeney@monash.edu (M.N.S.); 2 Nuclear Signaling Laboratory, Monash Biomedicine Discovery Institute and Department of Biochemistry and Molecular Biology, Monash University, Clayton, VIC 3800, Australiadavid.jans@monash.edu (D.A.J.)

**Keywords:** DDX3X, nuclear export, exportin-1, CRM1, NES, antiviral immunity, parainfluenza virus

## Abstract

DEAD-box helicase 3, X-linked (DDX3X) regulates the retinoic acid-inducible gene I (RIG-I)-like receptor (RLR)-mediated antiviral response, but can also be a host factor contributing to the replication of viruses of significance to human health, such as human immunodeficiency virus type 1 (HIV-1). These roles are mediated in part through its ability to actively shuttle between the nucleus and the cytoplasm to modulate gene expression, although the trafficking mechanisms, and impact thereof on immune signaling and viral infection, are incompletely defined. We confirm that DDX3X nuclear export is mediated by the nuclear transporter exportin-1/CRM1, dependent on an N-terminal, leucine-rich nuclear export signal (NES) and the monomeric guanine nucleotide binding protein Ran in activated GTP-bound form. Transcriptome profiling and ELISA show that exportin-1-dependent export of DDX3X to the cytoplasm strongly impacts IFN-β production and the upregulation of immune genes in response to infection. That this is key to DDX3X’s antiviral role was indicated by enhanced infection by human parainfluenza virus-3 (hPIV-3)/elevated virus production when the DDX3X NES was inactivated. Our results highlight a link between nucleocytoplasmic distribution of DDX3X and its role in antiviral immunity, with strong relevance to hPIV-3, as well as other viruses such as HIV-1.

## 1. Introduction

DEAD-box helicase 3, X-linked (DDX3X) is a conserved ATP-dependent RNA helicase with various roles in RNA metabolism/gene expression, facilitated by localization in the cytoplasm or the nucleus. DDX3X is crucial in regulating innate antiviral immune responses initiated by the retinoic-acid-inducible gene I (RIG-I)-like receptors (RLRs) [1]. RLRs recognize cytoplasmic RNA derived from viruses such as hepatitis C (HCV), influenza A, human immunodeficiency virus type 1 (HIV-1) [2], and parainfluenza virus type 3 (hPIV-3) [3], a major cause of bronchiolitis, bronchitis, and pneumonia in children, the elderly, and immunocompromised, and a cause of significant mortality in hematopoietic stem cell transplant recipients [4,5]. Despite being an important respiratory pathogen with no regulatory-approved vaccine or antiviral available, little is known of the host factors that mediate the response to hPIV-3 infection.

Upon binding viral RNA, RIG-I mobilizes the RLR signaling cascade to upregulate expression of type-I interferons (IFN-I), IFN-α, and -β. Activated RIG-I associates with the MAVS (mitochondrial antiviral signaling) adaptor protein, ultimately leading to recruitment of the IKKε (I-kappa-B kinase-ε) and TBK1 (TANK-binding kinase 1) complex. The IKKε/TBK1 complex, of which DDX3X is a crucial component [6,7,8], leads to the phosphorylation and nuclear translocation of interferon regulatory factors (IRF) 3 and 7 to activate IFN-I transcription [9]. In turn, IFN-I stimulates cognate receptors in an autocrine and paracrine manner to activate the Janus kinase/signal transducers and activators of transcription (JAK/STAT) signaling pathway, leading to the expression of hundreds of IFN-stimulated genes (ISGs) and driving both infected cells and nearby tissues to an antiviral state. DDX3X’s role in the cytosolic IKKε-TBK1 complex is to synergize with TBK1 to stimulate the *IFNB1* gene promoter, and also to bind directly to the promoter in the nucleus to enhance transcriptional activity [10]. With roles in both the nucleus and the cytoplasm, DDX3X nuclear trafficking is clearly paramount to its function.

The unmodified, monomeric form of DDX3X (73.2 kDa) is too large for efficient passive diffusion through the nuclear pore [11] and thus requires signal-dependent interaction with members of the importin/exportin family of nuclear transport proteins. Exportin-1 (also known as CRM1, chromosome region maintenance 1) is the primary nuclear export receptor for cargo proteins containing a leucine-rich nuclear export signal (NES) [12,13]. Guanosine triphosphate (GTP)-bound Ran (Ran-GTP) facilitates nuclear export by binding exportin-1 in the nucleus to allosterically promote cargo binding, while hydrolysis by Ran of GTP to GDP (guanosine diphosphate) in the cytosol dissociates exportin-1 from the translocated cargo [12,14]. Exportin-1-dependent nuclear export can be specifically inhibited by leptomycin B (LMB), which covalently binds the NES-binding groove of exportin-1 to block binding of NES-bearing cargo proteins in the nucleus [15,16]. Previous studies indicate the nuclear export of DDX3X is sensitive to LMB treatment [8,17,18,19,20], requires an N-terminal NES [20], and is exportin-1-dependent [20]. However, previous work identified a potential second export activity localized to the DDX3X helicase domain that was independent of either a conventional NES or Ran-GTP [17,21,22]. This activity was further reported to be utilized by HIV-1 for Rev-mediated nuclear export of retroviral response element (RRE)-containing unspliced or partially unspliced viral transcripts, thus is crucial for viral replication [17].

Here, we confirm that exportin-1-mediated nuclear export of DDX3X is dependent upon both an N-terminal NES and Ran-GTP. We further validate nuclear export-deficient DDX3X as a tool to establish the importance of exportin-1-dependent nuclear export in DDX3X’s various nuclear and cytosolic functions in the context of hPIV-3 infection. Using confocal laser scanning microscopy (CLSM) and, for the first time, analytical ultracentrifugation, we show that the association between exportin-1 and DDX3X is typical of other receptor–cargo interactions and that the leucine-rich NES recognized by exportin-1 is localized to residues 12–21. hPIV-3 infection or poly(I:C) transfection induces rapid DDX3X relocalization to the nucleus, underscoring the dynamic nature of DDX3X localization in response to invasive RNA. DDX3X in which the NES sequence is inactivated was localized more strongly in the nucleus and resulted in increased IFN-β secretion, and unexpectedly, elevated hPIV-3 virus production. LMB treatment, which blocks the nuclear export of all NES-bearing cargo, including DDX3X, suppressed hPIV-3 replication. This suggests trafficking of host/viral factors via exportin-1 is more predictive of hPIV-3 replicative fitness than IFN-β expression levels, and compounds targeting the nuclear export of unknown host and/or hPIV-3 viral proteins may be effective against hPIV-3. Finally, using NanoString RNA profiling we show the nucleocytoplasmic trafficking of DDX3X is critically important in regulating gene induction during viral infection, with marked differences in the ISG subset activated. Our findings have clear implications for the development of antivirals against viruses that utilize DDX3X as an essential host factor and in a compartment-specific manner, such as HIV-1 and respiratory syncytial virus (RSV).

## 2. Materials and Methods

### 2.1. Cell Lines

Human embryonic kidney epithelium (HEK-293T; female), human cervical epithelium adenocarcinoma (HeLa; female), and human alveolar epithelium adenocarcinoma (A549; male) cells were cultured in Dulbecco’s Modified Eagle Medium (DMEM; Thermo Fisher Scientific, Waltham, MA, USA), while African green monkey kidney epithelium (Vero; female) cells were cultured in DMEM-F12 (Thermo Fisher Scientific, Waltham, MA, USA). Both formulations were supplemented with 10% (*v/v*) FCS (Assay Matrix) and 2 mM L-glutamine and 100 U mL^−1^ penicillin/100 µg mL^−1^ streptomycin (Thermo Fisher Scientific, Waltham, MA, USA). Cells were maintained in a humidified cell incubator at 37 °C with 5% CO_2_. FACS-sorted cells were maintained in medium further supplemented with 100 μg mL^−1^ G-418 (Sigma-Aldrich, St. Louis, MO, USA) and discarded after 3 passages. All cells were negative for mycoplasma as determined by routine PCR and visualization of DNA counterstaining by confocal microscopy.

### 2.2. Molecular Cloning

Full-length human DDX3X and truncations were amplified from vector pCMV6-XL5-DDX3X and subcloned into pmCherry-C1 (*Eco*RI/*Kpn*I), pcDNA3.1-HA (*Kpn*I/*Eco*RI) or pcDNA3.1-myc-BirA* (*Not*I/*Bam*HI) for mammalian expression, or pCOLD-IV (*Nde*I/*Kpn*I) with sequence specifying an N-terminal 6His tag for bacterial expression. Full-length human exportin-1 and Ran(Q69L) were cloned into bacterial expression vector pGEX-6P immediately downstream of sequence encoding a PreScission protease cleavable GST-tag as previously described [23]. Site-directed mutagenesis was performed using the QuikChange method and the modifications confirmed by sequencing.

### 2.3. hPIV-3 Infection and Plaque Assays

Vero cells were used for the cultivation of a clinical strain of hPIV-3 (GenBank accession no. AY283063). For replicative fitness studies, A549 cells were infected with hPIV-3 at a multiplicity of infection (MOI) of 0.3 or 0.6. Cytopathic effects were monitored over the infection time course, and the cell-free virus was harvested 4–7 days postinfection by centrifugation at 4000 rcf for 5 min, then used to inoculate Vero cells to determine the virus titer by plaque formation. Plaque overlay was a 1:1 mix of 1.6% (*w/v*) SeaPlaque™ Agarose (Lonza, Rockland, ME, USA) and 2 × Leibovitz’s L15 medium (Thermo Fisher Scientific, Waltham, MA, USA), supplemented with 10% (*v/v*) FCS (Assay Matrix, Ivanhoe North, Victoria, Australia), 4 mM L-glutamine, 200 U mL^−1^ penicillin/200 µg mL^−1^ streptomycin (Thermo Fisher Scientific, Waltham, MA, USA), 40 mM HEPES pH 7.2 (Thermo Fisher Scientific, Waltham, MA, USA), 5 mL 7.5% (*v*/*v*) NaHCO_3_ (Sigma-Aldrich, St. Louis, MO, USA), and 0.5 ug mL^−1^ Amphotericin B (Thermo Fisher Scientific, Waltham, MA, USA). Cells were fixed 4–7 days postinfection using 1% (*w*/*v*) formal saline. Plaques were developed using 1 mL 0.1% (*w/v*) neutral red (Sigma-Aldrich, St. Louis, MO, USA).

### 2.4. DNA Transfections

Plasmid transfections were performed using FuGENE HD (Promega, Madison, WI, USA) according to the manufacturer’s instructions.

### 2.5. Cell Sorting

Cells were transfected to express mCherry-fused DDX3X(1–662), DDX3X(1–662)(qmNESα), or mCherry alone. Subconfluent cells were resuspended in D-PBS + 2% (*v/v*) FCS (Assay Matrix, Ivanhoe North, Victoria, Australia), then viable cells expressing each transgene were sorted using an Influx Cell Sorter (BD Biosciences, Franklin Lakes, NJ, USA; FlowCore, Monash University). Cell populations with introduced transgenes were maintained under 100 μg mL^−1^ G-418 antibiotic selection (Thermo Fisher Scientific, Waltham, MA, USA).

### 2.6. Co-Immunoprecipitation and Immunoblotting

HEK-293T cells were transfected to express mCherry or mCherry fusion proteins, then the mCherry positive populations were FACS-sorted and expanded. 1 × 10^7^ cells were scraped into microfuge tubes, then 200 μL ice-cold co-IP buffer (20 mM Tris-Cl pH 7.4, 150 mM NaCl, 0.1% *v/v* IPEGAL) supplemented with 10 μg mL^−1^ RNaseA (Sigma-Aldrich, St. Louis, MO, USA) and cOmplete Ultra EDTA-free protease inhibitor cocktail tablets was added. Cells were briefly sonicated and clarified by centrifugation, then 100 μL Protein G-coupled magnetic resin (Thermo Fisher Scientific, Waltham, MA, USA) pre-bound to mCherry antibody was added to each supernatant. Protein–antibody complexation proceeded with end-over agitation for 30 min at 4 °C, then the resin was washed once with tris-buffered saline (TBS), transferred to clean tubes, and 50 μL 2× Laemmli sample buffer was added before incubating for 10 min at 95 °C. Samples were centrifuged at 16,100× *g* for 1 min and immediately subjected to SDS-PAGE (sodium dodecyl sulfate polyacrylamide gel electrophoresis) on 10% polyacrylamide gels. Proteins were transferred to PVDF (polyvinylidene difluoride) and probed using specific antibodies diluted in 5% *w/v* skim milk + 0.1% *v/v* Tween-20.

### 2.7. NanoString RNA Profiling

Whole-cell lysates of freshly sorted A549 cells were prepared 24 h postinfection by thoroughly washing and resuspending the cells in 50 μL CL buffer (10 mM Tris-Cl pH 7.4, 150 mM NaCl, 0.25% (*v/v*) IPEGAL). Cells were homogenized and RNA hybridization reactions were performed using the 770-plex Human PanCancer Immune Profiling CodeSet (NanoString Technologies, Seattle, WA, USA) with 5 μL clarified supernatant, corresponding to approximately 4000 cells in accordance with the manufacturer’s instructions. The nCounter^®^ SPRINT system (NanoString Technologies, Seattle, WA, USA) was used to quantify captured reporter probes. Average linkage Pearson correlation heatmaps on optimally ordered data were generated using MeV software. Principal component analysis was performed using XLStat. Experimentally-validated human ISGs were interrogated using the Interferome database [24].

### 2.8. Enzyme-Linked Immunosorbent Assay

ELISA titrations were performed in triplicate using the sandwich method employed by the LumiKine hIFN-β kit (Invivogen, San Diego, CA, USA) in accordance with the manufacturer’s instructions. Measurements were performed on a ClarioStar plate reader (BMG Labtech, Ortenberg, Germany) equipped with a liquid injector using 30 flashes per well.

### 2.9. Immunocytochemistry

Cells were fixed with 4% (*w/v*) paraformaldehyde in PBS 24–48 h post-transfection. Specimens were permeabilized and blocked with 5% (*w/v*) BSA in PBS + 0.25% (*v/v*) Triton X-100 (Sigma-Aldrich, St. Louis, MO, USA) overnight at 4 °C, then incubated with primary antibodies diluted in 1% (*w/v*) BSA in PBS + 0.1% (*v/v*) Triton X-100. Cells were washed extensively, then diluted secondary antibodies were added and incubated for 2 h. 1 mM Hoechst 33342 (Thermo Fisher Scientific, Waltham, MA, USA) was added for nuclear counterstaining. Specimens were mounted with Dako antifade mounting medium (Agilent, Santa Clara, CA, USA).

### 2.10. Live and Indirect Immunofluorescence Microscopy

Fixed and live cell imaging was performed using a Nikon C1 inverted confocal laser scanning microscope (Monash Micro Imaging, Monash University), equipped with a CO_2_- and temperature-controlled live imaging chamber and stage, a 100× NA 1.4 oil-immersion objective, and running NIS Elements (Nikon, Tokyo, Japan) for image acquisition. Specimens were optically sliced through the maximum dimension of the nucleus using a pinhole diameter of 1.0 AU. Images were analyzed blind using Fiji/ImageJ (NIH). Pixel intensities (fluorescence) in the middle of the nucleus and cytosol were determined by sampling equally sized representative regions of interest (ROIs), free of inclusions and oversaturated pixels, as performed previously [25]. Background was calculated by defining a ROI in each image lacking cells or specific staining, and measuring the pixel intensity of an area equivalent to that used for cell sampling. This was subtracted from the nuclear and cytosolic pixel intensity values, thereby enabling the nuclear/cytoplasmic (Fn/c) ratio to be calculated. Similarly, DDX3X-exportin-1 colocalization around the nuclear membrane was determined by measuring pixel intensity (fluorescence) along a representative line bisecting the nucleocytosolic boundary as indicated. 

### 2.11. Expression and Purification of DDX3X

p-COLD-6His-DDX3X(1-580) wild-type and NES mutants were expressed in *Escherichia coli* BL21(DE3) cells at 16 °C following induction at OD_600nm_ = 0.6 with 1 mM isopropyl 1-thiol-β-D-galactopyranoside (IPTG; Astral Scientific, NSW, Australia). 20 h post-induction, bacteria were harvested by centrifugation and resuspended in lysis buffer [20 mM Tris-Cl pH 8.0, 500 mM NaCl, 20% (*v/v*) glycerol, 0.5 mM TCEP, 10 mM imidazole, supplemented with 1 mg mL^−1^ lysozyme (Astral Scientific, NSW, Australia), 0.1% (*v/v*) Tween-20 and cOmplete protease inhibitor tablets (Sigma-Aldrich, St. Louis, MO, USA)]. Proteins were extracted by sonication and clarified by centrifugation, then applied to Ni-NTA Superflow resin (Qiagen, Hilden, Germany). Resin was washed with lysis buffer + 20 mM imidazole and 1% (*v/v*) Tween-20, then 6His-DDX3X was eluted with lysis buffer + 300 mM imidazole. Eluate was then applied to a Superdex 200 16/60 gel filtration column equilibrated in DDX3X SEC buffer (20 mM Tris-Cl pH 8.0, 500 mM NaCl, 20% (*v/v*) glycerol, 0.5 mM TCEP), then fractions containing the purest monomeric 6His-DDX3X(1-580) were pooled.

### 2.12. Expression and Assembly of Exportin-1-Ran-GTP

GST-Exportin-1 and GST-Ran(Q69L) were expressed separately in *E. coli* BL21(DE3) cells at 16 °C following induction at OD_600nm_ = 0.6 with 0.5 mM IPTG. 18 h post-induction, bacteria were harvested by centrifugation and resuspended in PBS supplemented with 5 mM DTT and cOmplete protease inhibitor tablets (Sigma-Aldrich, St. Louis, MO, USA). Proteins were extracted by sonication and applied to sepharose G4B resin (GE Healthcare, Chicago, IL, USA) for GST-affinity purification, then washed and the GST-free proteins eluted by incubation with PreScission protease. Exportin-1 and Ran(Q69L) were then each applied to a Superdex 200 16/60 gel filtration column (GE Healthcare, Chicago, IL, USA) equilibrated in GF1 buffer (20 mM Tris-Cl pH 7.5, 100 mM NaCl, 5 mM MgOAc, 2 mM DTT). For production of Ran-GTP, 1 mM GTP was added to Ran(Q69L) and the complex was purified on a Superdex 200 16/60 column. The formation of the Ran-GTP complex was confirmed by absorbance at 260 nm. For binding studies using exportin-1-Ran-GTP, the complex was pre-formed by incubating equimolar amounts of exportin-1 and Ran-GTP in GF1 Buffer at 20 °C for 30 min.

### 2.13. Circular Dichroism

Protein circular dichroism spectra were measured in 20 mM Tris-Cl pH 8.0, 500 mM NaCl, 10% glycerol, and 0.5 mM TCEP using a J-815 circular dichroism (CD) spectrometer (Jasco, Easton, MD, USA). Spectra were recorded at 0.2 mg mL^−1^ between 190–250 nm in a 1 mm quartz cuvette at 20 °C. Mean ellipticity values per residue (*θ*) were calculated as *θ =* (3300 × *m* × Δ*A)*/(*lcn*), where *l* is the path length (0.1 cm), *n* is the number of residues, *m* is the molecular mass (Da), and *c* is the protein concentration (mg mL^−1^).

### 2.14. RNA-Dependent ATP Hydrolysis Assays

RNA-dependent ATP hydrolysis activity was measured using the Biomol^®^ Green phosphate detection kit (Enzo Life Sciences, Farmingdale, NY, USA). 200 nM RIG-IΔCARDS, DDX3X, or variants thereof were diluted in ATPase assay buffer (20 mM Tris-Cl pH 7.5, 1.5 mM DTT, 1.5 mM MgCl_2_), 10 μM poly(I:C) (Invivogen, San Diego, CA, USA), and 20 nmol ATP (Sigma-Aldrich, St. Louis, MO, USA), then incubated for 25 min at 37 °C. Phosphate standards were serially diluted from 2 μM to 0.031 μM using 1× ATPase reaction buffer and added to the control wells. Reactions were performed in pentaplicate in a final volume of 100 μL in 96-microwell assay plates (Corning, Corning, NY, USA). Reagents were diluted using diethylpyrocarbonate (DEPC)-treated water. Following incubation, 100 μL Biomol Green reagent was added to the control and sample wells to stop the reactions. Sample absorbance was measured by absorbance at 620 nm using a ClarioStar plate reader (BMG Labtech, Ortenberg, Germany) using 30 flashes per well.

### 2.15. Analytical Ultracentrifugation

Sedimentation velocity experiments on wild-type DDX3X and NES mutants alone and in complex with exportin-1-Ran-GTP were performed in an Optima analytical ultracentrifuge (AUC; Beckman Coulter, Brea, CA, USA) at 20 °C. Proteins were incubated individually or together at 20 °C for 30 min prior to centrifugation in GF1 buffer (20 mM Tris-Cl pH 7.5, 100 mM NaCl, 5 mM MgOAc, 2 mM DTT). 380 μL of sample and 400 μL of reference solution (GF1 buffer) were loaded into a conventional double sector quartz cell and mounted in an An-50 Ti rotor (Beckman Coulter, Brea, CA, USA). Samples were centrifuged at 40,000 rpm and the data was collected continuously at 280 nm. Solvent density (1.041 g mL^−1^ at 20 °C) and viscosity (1.0149 cp at 20 °C), as well as estimates of the partial specific volume (DDX3X: 0.7215 mL g^−1^, exportin-1-Ran-GTP: 0.7450 mL g^−1^ at 20 °C), were computed using SEDNTERP [26]. Sedimentation velocity data were fitted to a continuous size [*c*(*s*)] distribution model using SEDFIT [27].

### 2.16. Quantification and Statistical Analysis

**Figure 1:**
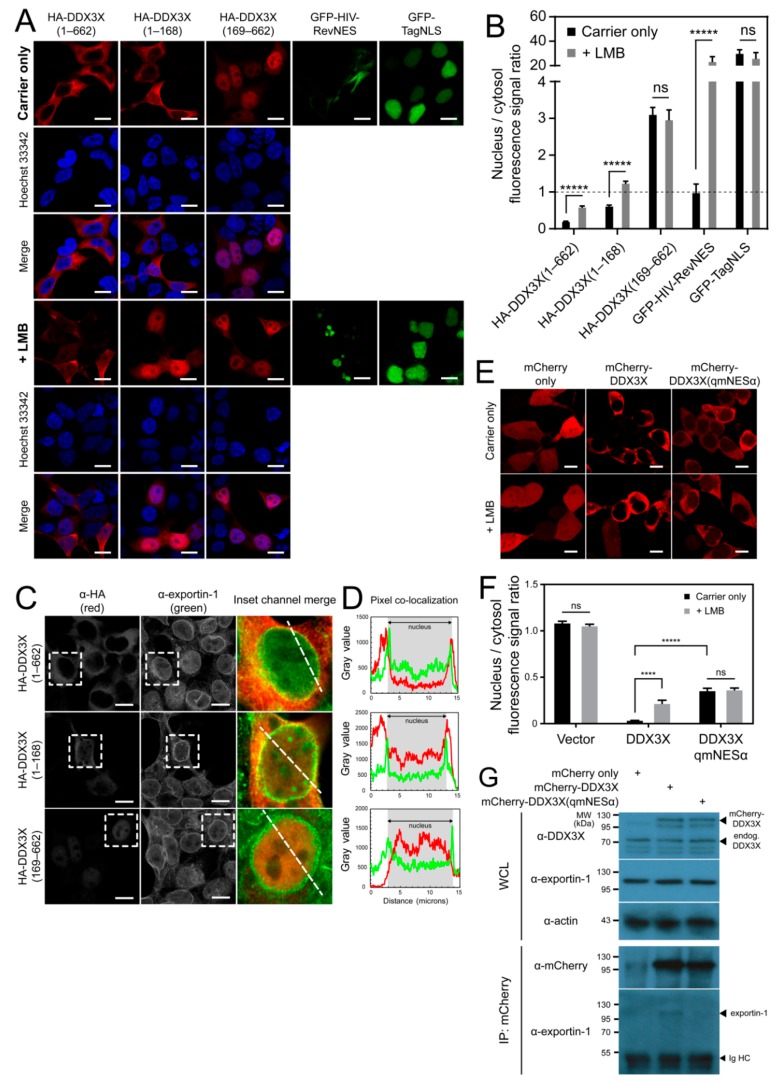
The DEAD-box helicase 3, X-linked (DDX3X) N-terminal region mediates nuclear export by exportin-1. (**A**) Human embryonic kidney (HEK)-293T cells transfected to express HA-DDX3X(1‒662), HA-DDX3X(1‒168), HA-DDX3X(169‒662), GFP-HIV-RevNES or GFP-TagNLS, treated 24 h post-transfection with 20 nM Leptomycin B (LMB) or carrier only (EtOH) for 6 h, then fixed, immunostained using α-HA antibody and DNA counterstained using Hoechst 33342, then visualized by CLSM. Representative cell clusters and channel-merged images shown. Scale bar = 10 μm. (**B**) Images including those in panel A were analyzed to determine the nucleus/cytosol fluorescence signal ratio. Results represent the mean ±SEM (*n* = 50). Student’s *t*-test with Holm–Sidak multiple comparisons test for significance; *****, *p* ≤ 0.0001; ****, *p* ≤ 0.001; ns, not significant. (**C**) HEK-293T cells transfected to express HA-DDX3X(1–662), HA-DDX3X(1–168) or HA-DDX3X(169–662), then fixed and immunostained using α-HA (red channel) or α-exportin-1 (green channel) antibodies and visualized by CLSM. Scale bar = 10 μm. Representative cell clusters shown. White dotted box denotes zoomed and channel-merged area; white dashed line therein denotes area of pixel intensity measurement shown in panel D. (**D**) Pixel intensity measurements of cells overexpressing HA-DDX3X variants in panel C. Nuclear membrane boundaries are shown graphically by green channel pixel intensity maxima and highlighted by grey shading, validated with Hoechst 33342 DNA counterstain (not shown). (**E**) HEK-293T cells transfected to express mCherry-fused wild-type DDX3X or DDX3X(qmNESα), visualized by live CLSM. Representative cell clusters shown. Scale bar = 10 μm. (**F**) Images including those in panel E were analyzed to determine the nuclear-to-cytosolic fluorescence signal ratio (Fn/c). Results represent the mean ± SEM (*n* = 50). Student’s *t*-test with Holm–Sidak multiple comparisons test for significance; *****, *p* ≤ 0.0001; ****, *p* ≤ 0.001; ns = not significant. (**G**) FACS-sorted HEK-293T cells expressing full-length mCherry-DDX3X, mCherry-DDX3X(qmNESα) or mCherry only, lysed and subjected to anti-mCherry co-immunoprecipitation, then immunoblotted for mCherry-DDX3X–bound endogenous proteins. WCL = whole cell lysate; Ig HC = immunoglobulin heavy chain.

**Figure 2:**
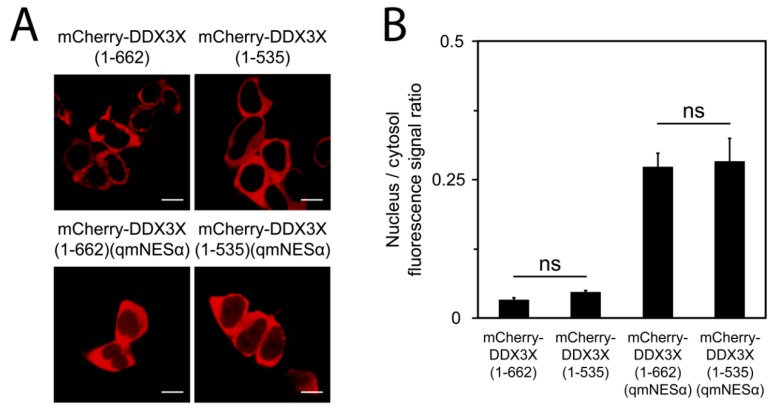
The C-terminal tail is dispensable for the nucleocytoplasmic distribution of DDX3X. (**A**) HEK-293T cells transfected to express mCherry-DDX3X(1–662) or mCherry-DDX3X(1–535), harboring a wild-type or defective NES, visualized by live CLSM. Scale bar = 10 μm. (**B**) Quantitation of the nuclear-to-cytosolic fluorescence signal ratio (Fn/c) of cells shown in panel A. Results represent the mean ± SEM (*n* = 50). Student’s *t*-test for significance with Holm–Sidak multiple comparisons test for significance; ns, not significant.

**Figure 3:**
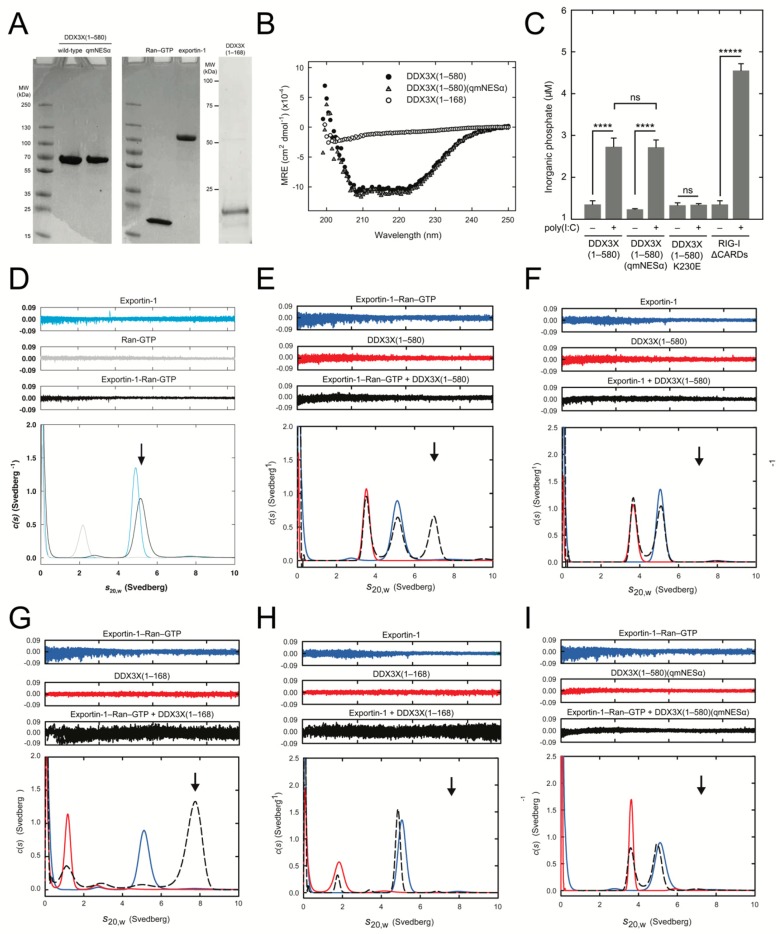
A nuclear export signal (NES) within the DDX3X N-terminal region is necessary and sufficient for direct binding to exportin-1 in a Ran-GTP-dependent manner. (**A**) Coomassie-stained SDS-PAGE of recombinant DDX3X(1–580), DDX3X(1–580)(qmNESα), Ran-GTP, exportin-1 and DDX3X(1–168). (**B**) Circular dichroism spectra of DDX3X(1–580) (black circles), DDX3X(1–580)(qmNESα) (grey triangles), and DDX3X(1–168) (white circles). Ellipticity (mdeg) normalized to mean residue ellipticity (deg cm^2^ dmol^−1^). (**C**) RNA-dependent ATP hydrolysis assay measuring inorganic phosphate production from the ATPase activity of recombinant wild-type DDX3X(1‒580) or variants thereof. The ATPase-deficient DDX3X(K230E) mutant was a negative control, while the RIG-IΔCARDS protein was a positive control. Results represent the mean ± SD (*n* = 3). Student’s *t*-test with Holm–Sidak multiple comparisons test for significance; *****, *p* ≤ 0.0001; ****, *p* ≤ 0.001; ns, not significant. (**D**) Continuous sedimentation coefficient distribution [*c*(*s*)] plotted as a function of *s*_20,w_ for recombinant exportin-1 (light blue line), Ran-GTP (gray line) and the exportin-1-Ran-GTP complex following incubation at equimolar concentrations (black line). (**E**–**H**) Continuous sedimentation coefficient distribution [*c*(*s*)] plotted as a function of *s*_20,w_ for recombinant DDX3X variants (red line) and exportin-1 with or without Ran-GTP (blue line) alone and following incubation at equimolar concentrations (black dashed line). For (**D**–**I**) residuals from the *c*(*s*) distribution best fit plotted as a function of radial distance from the axis of rotation are displayed above. The presence or absence of larger-sedimenting species corresponding to complex formation is indicated by black arrows. See also Table 1.

**Figure 4:**
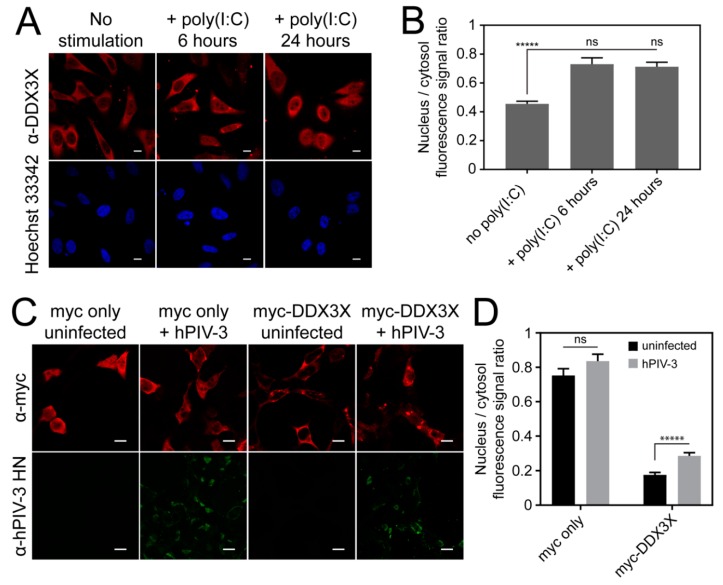
DDX3X nuclear localization increases during virus infection in response to invasive RNA. (**A**) Human cervical epithelium adenocarcinoma (HeLa) cells transfected with 20 μM poly(I:C) or carrier (H_2_O) for 6 or 24 h, then fixed and immunostained using α-DDX3X antibody, counterstained using Hoechst 33342 and visualized by CLSM. Representative cell clusters shown. (**B**) Quantitation of the nuclear-to-cytosolic fluorescence signal ratio (Fn/c) of cells including those shown in panel A. Results represent the mean ± SEM (*n* = 30). One-way ANOVA with Dunnett’s multiple comparisons test for significance. (**C**) A549 cells transfected to express myc-tagged-DDX3X or myc alone, infected with hPIV-3 at MOI = 0.3, fixed and immunostained using α-myc and α-hPIV-3 HN antibodies, then visualized by CLSM. Representative cell clusters shown. (**D**) Quantitation of the nuclear-to-cytosolic fluorescence signal ratio (Fn/c) of cells including those shown in panel C. Results represent the mean ± SEM (*n* = 30 cells). Student’s *t*-test with Holm–Sidak multiple comparisons test for significance; *****, *p* ≤ 0.0001; ns, not significant. Scale bars = 10 μm.

**Figure 5:**
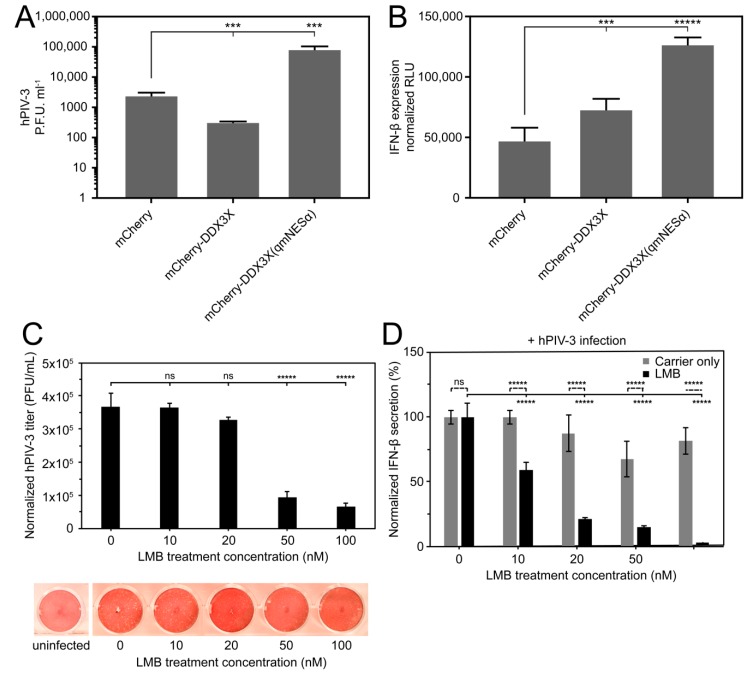
Nuclear export-defective DDX3X enhances IFN-β expression but also hPIV-3 replication. (**A**) Vero cell monolayer plaque assays measuring hPIV-3 replicative fitness (MOI = 0.3) in transfected A549 cells overexpressing mCherry-fused DDX3X, DDX3X(qmNESα), or mCherry alone. Results represent the mean ± SD (*n* = 3). One-way ANOVA with Dunnett’s multiple comparisons test for significance; ***, *p* ≤ 0.01. (**B**) Bioluminescent ELISA for secreted IFN-β in culture medium from A549 cells stably expressing mCherry-DDX3X, mCherry-DDX3X(qmNESα), or mCherry alone, infected with hPIV-3 (MOI = 0.3) for 5 days. Results represent the mean ± SD (*n* = 3). One-way ANOVA with Tukey’s all-column multiple comparisons test for significance; *****, *p* ≤ 0.0001; ***, *p* ≤ 0.01. (**C**) Vero cell monolayer plaque assays measuring hPIV-3 replicative fitness (MOI = 0.6) in A549 cells. Prior to infection, A549 cells were treated for 4 h with the indicated concentration of LMB, infected, then treated with this same concentration of LMB for 36 h, replenishing every 12 h during this period to account for the activity half-life and cellular turnover of exportin-1. Data were normalized to input protein levels as determined by Bradford assay on adherent whole cell lysates. Results represent the mean ± SD (*n* = 3). One-way ANOVA with Dunnett’s multiple comparisons test for significance. *****, *p* ≤ 0.0001; ns = not significant. Representative photomicrographs of plaques shown. (**D**) Bioluminescent ELISA for secreted IFN-β in culture medium from A549 cells. Culture medium from cells in panel C was collected, clarified by centrifugation, then assayed for secreted IFN-β by ELISA as in panel B. Results represent the mean ±SD (*n* = 4). One-way ANOVA with Tukey’s multiple comparisons test for significance; *****, *p* ≤ 0.0001; ns = not significant.

**Figure 6:**
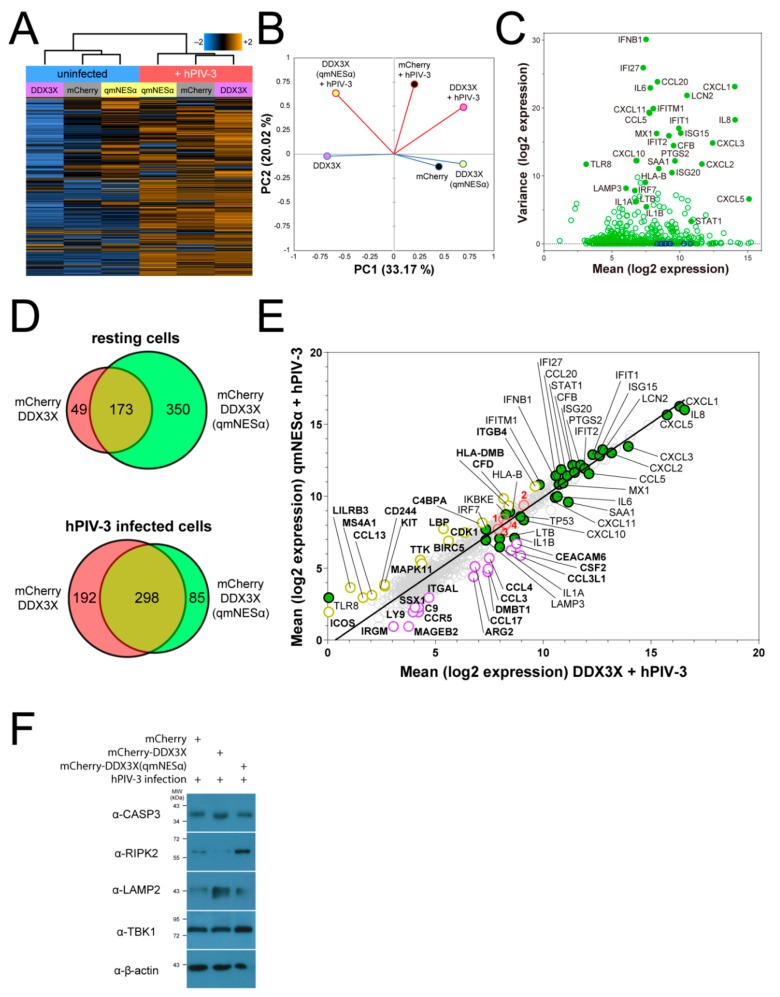
Lack of DDX3X nuclear export impacts steady-state and virus-induced transcription profiles and interferon-induced antiviral responses. NanoString transcriptional profiling was performed for A549 cells stably overexpressing mCherry-DDX3X, mCherry-DDX3X(qmNESα), or mCherry alone and infected with or without hPIV-3 for 24 h. (**A**) Cluster heatmap of Pearson correlation averaged log_2_-normalized levels of all 730 gene transcripts. Relative RNA levels are plotted as z-scores using the indicated color heatmap (blue = decrease, orange = increase, black = no change). (**B**) Pearson correlation principal component analysis of transcription data from panel A. Lines denote infection status (blue = uninfected, red = hPIV-3-infected) and filled circles denote protein-expressing populations (purple = mCherry-DDX3X, yellow = mCherry-DDX3X(qmNESα), black = mCherry only). (**C**) Scatterplot of normalized expression mean vs. variance of all genes in uninfected versus hPIV-3 infected vector only transfected cells. Gene expression was normalized against 10 stable housekeeping genes (*CNOT10*, *CNOT4*, *TBP*, *DDX50*, *NUBP1*, *FCF1*, *PRPF38A*, *NOL7*, *ABCF1*, and *EIF2B4*). Endogenous genes (green open circles) of interest with the greatest expression and/or variance labeled (green filled circles), together with stable housekeeping genes (blue open circles). (**D**) Venn diagram representation of the total number of genes in panel A upregulated by mCherry-DDX3X (red), mCherry-DDX3X(qmNESα) (green), or both (yellow) at rest or during hPIV-3 infection. (**E**) Scatterplot of log_2_-normalized expression mean of all genes in cells expressing DDX3X vs. DDX3X(qmNESα) during hPIV-3 infection. Line of best fit (black line) through all genes (gray open circles). Genes highly expressed during hPIV-3 infection in panel D are highlighted (green filled circles) and labelled. Besides these genes, top 15 differentially-transcribed genes are also highlighted (DDX3X, purple open circles; DDX3X(qmNESα), yellow open circles) and labelled in bold type. Genes whose encoded protein expression levels are validated in panel F are highlighted in red open circles and labelled in red bold type (1 = *TBK1*, 2 = *RIPK2*, 3 = *CASP3*, 4 = *LAMP2*). (**F**) FACS-sorted, hPIV-3-infected A549 cells expressing full-length mCherry-DDX3X, mCherry-DDX3X(qmNESα), or mCherry only, lysed and immunoblotted for CASP-3, RIPK2, LAMP2, or TBK1 expression.

Statistical parameters are reported in the figures and figure legends. Statistical analysis was performed using GraphPad Prism software. For nuclear/cytosolic fluorescence ratio measurements (Figures 1B,F, 2B and 4B,D), n represents the number of cells measured per sample and is represented as mean ± SEM, as previously [23,28,29]. Significance was calculated using Student’s *t*-test (two-tailed) or one-way ANOVA with Tukey’s, Dunnett’s, or Holm–Sidak multiple comparisons post hoc analysis as indicated. For ATP hydrolysis assays (Figure 3C), n represents the number of experimental replicates and is represented as mean ± SD. Significance was calculated using the Student’s *t*-test with Holm–Sidak multiple comparisons post hoc analysis. For plaque assays (Figure 5A,C) and ELISA (Figure 5B,D), n represents the number of biological replicates and is represented as mean ± SD. Significance was calculated using one-way ANOVA with Dunnett’s or Tukey’s multiple comparisons post hoc analysis as indicated. For NanoString RNA profiling (Figure 6A–C, Table S1), low (<10) count data was discarded, then the remaining data was background corrected by subtracting the maximum value of the available negative control probes and normalized to the geometric mean of 10 stable housekeeping genes across all samples, as described previously [30]. 

## 3. Results

### 3.1. The DDX3X N-Terminus Mediates Its Nuclear Export

To investigate the exportin-1-dependent nuclear export of DDX3X, we generated plasmids for mammalian expression of HA-fused full-length DDX3X, the first 168 residues (HA-DDX3X(1–168)), or DDX3X lacking the first 168 residues (HA-DDX3X(169–662)). Subcellular localization of these proteins was analyzed in HEK-293T cells by confocal laser scanning microscopy and quantitative image analysis (qCLSM). As a control, we used a GFP fusion of the well-characterized HIV-1 Rev NES (GFP-HIV-RevNES), which is exported via exportin-1 [31]. As another control, we used a GFP fusion of the Simian virus 40 T-antigen (Tag) NLS (GFP-TagNLS), which undergoes nuclear import dependent on importin α/β1 [32]. As expected, GFP-HIV-RevNES and GFP-TagNLS localized to the cytoplasm and nucleus, respectively (Figure 1A,B). Surprisingly, despite being only 19.5 kDa in size, and small enough in principal to passively diffuse across the nuclear pore, HA-DDX3X(1–168) was predominantly cytosolic, as per the full-length protein (Figure 1A,B). In contrast, DDX3X lacking this region, HA-DDX3X(169–662), was localized strongly within the nucleus (Figure 1A,B), supporting the idea that the N-terminus specifically mediates nuclear export of DDX3X [20]. Additionally, because the 55.9 kDa HA-DDX3X(169–662) truncation has very limited ability to passively diffuse through the nuclear pore, its strong nuclear accumulation is likely due to active nuclear import. Thus, DDX3X residues 1–168 and 169–662 appear to harbor, respectively, at least one NES or nuclear localization signal (NLS), and these interact specifically with one or more subcellular trafficking receptors to facilitate nucleocytoplasmic shuttling of full-length DDX3X.

### 3.2. Exportin-1 Is a DDX3X Nuclear Export Receptor

To further investigate the nuclear trafficking of DDX3X, we compared the sensitivity of full-length DDX3X(1–662) and the 1–168 and 169–662 DDX3X fragments to LMB, a specific inhibitor of exportin-1-mediated nuclear export. As expected, LMB treatment significantly (*p* ≤ 0.0001) inhibited nuclear export of GFP-HIV-RevNES (24-fold higher nuclear accumulation) and had no effect on nuclear localization of GFP-TagNLS (*p* = 0.5063) (Figure 1A,B), ruling out nonspecific effects. LMB treatment caused significantly (*p* ≤ 0.0001) increased nuclear accumulation of full-length DDX3X (~3-fold), consistent with previous reports [8,17,18,19], as well as the 1–168 fragment (~2-fold), but not the 169–662 fragment (Figure 1A,B). These results suggest that residues 169–662 do not confer exportin-1 mediated trafficking. Consistent with these results, we also observed extensive colocalization of endogenous exportin-1 and exogenous full-length DDX3X or DDX3X(1–168), but not DDX3X(169–662), in structures consistent with nuclear pores around the nuclear membrane (Figure 1C,D). Thus, our data show that DDX3X residues 1-168 confer exportin-1-dependent nuclear export, consistent with the identification of a NES recognized by exportin-1 within the first 22 residues of DDX3X [20]. 

### 3.3. DDX3X Harbors an Exportin-1 Recognized NES in the N-Terminus

To confirm the NES (hereafter termed NESα) is functional in mediating DDX3X nuclear export, and for further use as a tool to explore DDX3X function, we substituted the four hydrophobic residues to alanine (L12A/F16A/L19A/L21A, termed qmNESα), which are required for transport of other exportin-1 cargos [33]. We compared subcellular distribution of the qmNESα variant with wild-type DDX3X expressed as mCherry fusion proteins in HEK-293T cells by live-cell qCLSM. The qmNESα DDX3X variant showed significantly (*p* ≤ 0.0001) impaired nuclear export compared to wild-type, with almost 9-fold higher levels of nuclear accumulation (Figure 1E,F). LMB treatment did not further increase the nuclear fluorescence signal, in stark contrast to wild-type which showed significantly (*p* ≤ 0.001) increased nuclear accumulation. As expected, these results confirmed the finding by Brennan et al. [20] that the DDX3X N-terminal NESα is functional in exportin-1-dependent nuclear export (Figure 1E,F). Consistent with this result, we successfully captured the transient receptor–cargo interaction between endogenous exportin-1 and mCherry-DDX3X, but not mCherry-DDX3X(qmNESα) by co-immunoprecipitation (Figure 1G). Collectively, these data confirm that exportin-1-mediated nuclear export of DDX3X is dependent on the N-terminal NESα of DDX3X. 

### 3.4. DDX3X’s C-Terminal Tail Is Dispensable for Nuclear Export

DDX3X residues 260–517, comprising a truncated portion of the helicase core (residues 211–575), were previously proposed to bind exportin-1 without dependence on a modular NES- or Ran-GTP [17,21,22]. Additionally, DDX3X C-terminal residues 536–662 have been reported to mediate nuclear export by nuclear RNA export factor 1 (NXF1/TAP) [18]. To test these possibilities, we generated mCherry-fused DDX3X lacking the NXF1-binding region but harboring the wild-type NESα, termed DDX3X(1–535). Using live-cell qCLSM, we found the subcellular distribution of this protein was identical to full-length (Figure 2), indicating the C-terminal tail is dispensable for DDX3X’s subcellular trafficking. Next, we introduced our qmNESα mutations into this truncated construct, termed DDX3X(1–535)(qmNESα), to test the contribution of any NES-independent binding of exportin-1. As expected, this protein was localized in an identical manner to full-length DDX3X(qmNESα) (Figure 2). Collectively, these data suggest DDX3X’s bulk nuclear export occurs via exportin-1, and that this is mediated by the N-terminal NESα-sequence.

### 3.5. DDX3X-exportin-1 Binding Requires Ran-GTP

To confirm Ran-dependence of binding of exportin-1 to the DDX3X NESα in a direct manner, we performed AUC sedimentation velocity experiments using a minimally reconstituted system. We produced purified recombinant exportin-1, Ran-GTP, DDX3X(1-168), DDX3X(1–580), and DDX3X(1–580)(qmNESα) (Figure 3A), then validated correct folding of each DDX3X variant by circular dichroism (Figure 3B). As a further quality control, we confirmed that the RNA-dependent ATPase activity of DDX3X(1–580)(qmNESα) was identical to wild-type (Figure 3C), in contrast to an ATPase deficient DDX3X mutant (K230E), thus confirming that the mutations in the qmNESα variant do not disrupt the overall conformation of DDX3X or its function.

Following sedimentation velocity experiments, *c*(*s*) analysis revealed exportin-1, exportin-1-Ran-GTP, DDX3X(1–580), and DDX3X(1–168) all sediment as single species with sedimentation coefficients (*s*_20,w_) of 4.9S, 5.1S, 3.6S, and 1.7S, respectively, consistent with their molecular weights (Figure 3D–I and Table 1). When exportin-1 was combined with Ran-GTP we observed a 0.2S difference between the exportin-1-Ran-GTP complex (5.1S) and exportin-1 alone (4.9S), suggesting the exportin-1-Ran-GTP complex has formed (Figure 3D and Table 1). When we combined DDX3X(1–580) in a 1:1 molar ratio with exportin-1-Ran-GTP, we observed a larger 7.0S species consistent with the molecular weight of a DDX3X-exportin-1-Ran-GTP complex (Figure 3E and Table 1). Crucially, this was not detected in the absence of Ran-GTP (Figure 3F) and, as expected, we observed a similar pattern when we substituted DDX3X(1–580) for the DDX3X(1–168) fragment (Figure 3G,H and Table 1). Notably, when present, there was no evidence of free Ran-GTP (2.2S), suggesting complete formation of the exportin-1-Ran-GTP complex. Finally, we tested the specific requirement of the DDX3X NESα motif for exportin-1 binding. DDX3X(1–580)(qmNESα) sedimented as a single species with a *s*_20,w_ of 3.7S, and did not form a larger-sedimenting species when combined with exportin-1-Ran-GTP (Figure 3I and Table 1), confirming that the DDX3X NESα motif is required for direct binding by exportin-1. Taken together, our results confirm a direct, physiologically relevant interaction between exportin-1 and DDX3X that is explicitly NESα- and Ran-GTP-dependent.

### 3.6. Invasive RNA Triggers DDX3X Nuclear Accumulation

We next probed the functional significance of exportin-1-dependent nuclear export of DDX3X in innate immune signaling in the context of invasive RNA. To determine whether the subcellular distribution of DDX3X changes in correlation with immune stimulation, we challenged HeLa cells by transfection with the synthetic double-stranded RNA analog poly(I:C) and then examined the subcellular distribution of endogenous DDX3X by qCLSM. Strikingly, poly(I:C) caused rapid redistribution of DDX3X from the cytosol to the nucleus, with significantly (*p* ≤ 0.0001) increased (~2-fold) nuclear accumulation observed 6 h post-stimulation, with levels of nuclear protein remaining constant for at least 24 h (Figure 4A,B). To test whether the same effects were induced by an RNA virus infection model, we used hPIV-3, the most virulent hPIV subtype for respiratory illness [34], and A549 human alveolar epithelial cells. Indeed, hPIV-3 infection significantly (*p* ≤ 0.0001) increased (~2-fold) the nuclear localization of ectopically expressed DDX3X (Figure 4C,D). Notably the magnitude of DDX3X relocalization between poly(I:C) stimulation and virus infection was identical (~2-fold), suggesting a specific response to invasive RNA. In addition, hPIV-3 infection induced accumulation of DDX3X into cytosolic inclusions in some cells, possibly p-bodies or stress granules typically associated with translational regulation of cellular or viral RNA. HeLa cells showed identical results (data not shown). These results imply that nuclear redistribution of DDX3X may be a general, acute-phase cellular response to viral challenge, arising as a specific cellular response to invasive RNA.

### 3.7. Overexpression of Wild-Type But Not Nuclear Export Defective DDX3X Can Protect Against hPIV-3 Infection

To dissect the role of exportin-1-mediated nuclear export of DDX3X in regulating immune signaling events in the nucleus and cytosol, we infected A549 cells expressing either mCherry-DDX3X, mCherry-DDX3X(qmNESα), or mCherry alone with hPIV-3, and then measured viral replicative fitness using plaque assays. Strikingly, cells overexpressing mCherry-DDX3X were significantly (*p* ≤ 0.01) more resistant to infection than those expressing mCherry alone, with almost a 10-fold reduction in infectious virus production as measured by plaque assay (Figure 5A). This is consistent with the idea that DDX3X plays an important antiviral role. In stark contrast, cells overexpressing mCherry-DDX3X(qmNESα) were substantially more susceptible to infection, with 200-fold higher levels of virus production (*p* ≤ 0.01) than those expressing wild-type DDX3X, strongly indicating that DDX3X’s ability to undergo nuclear export through the exportin-1-recognized NESα is key to its antiviral activity. Parallel monitoring of production of IFN-β by ELISA in response to infection indicated that DDX3X(qmNESα)-expressing cells secreted significantly (*p* ≤ 0.001) more IFN-β (~2-fold) than those expressing wild-type DDX3X (Figure 5B). These results suggest that the increased hPIV-3 titer observed in nuclear export defective DDX3X-expressing cells is not due to a general defect in IFN-β production during hPIV-3 infection, and that increased IFN-β production is insufficient to inhibit hPIV-3 replication. These results strongly imply that DDX3X’s antiviral role in hPIV-3 infection is dependent on its nuclear export/nuclear trafficking ability.

### 3.8. Exportin-1 Is Important to hPIV-3 Replication

Even though RNA viruses such as paramyxoviruses replicate entirely in the host cytosol, inhibition of exportin-1 by LMB has been reported to inhibit virus production in the case of Hendra virus [23], RSV [35], and Venezuelan equine encephalitis virus [36], suggesting their replication is facilitated by exportin-1-dependent nuclear export. Since hPIV-3 also replicates entirely in the cytoplasm, we tested the importance of exportin-1 mediated nuclear export by treating hPIV-3-infected cells with LMB, again monitoring virus production and IFN-β as above. Controlling for limited cytotoxic effects, we found a dose-dependent reduction in both hPIV-3 titer and IFN-β secretion with increasing LMB concentration (Figure 5C,D), again consistent with the importance of exportin-1-dependent nuclear export of host/viral factors being central to hPIV-3 virus production fitness, as opposed to IFN-β levels.

### 3.9. DDX3X’s Nuclear Trafficking Potentiates Immune Gene Induction

Since IFN-β production in response to hPIV-3 infection did not appear to be impaired by inactivation of DDX3X nuclear export, we hypothesized that altered expression of antiviral genes besides *IFNB1* might be responsible for the effects on infection observed in Figure 4A. To address this directly, we profiled host gene transcription using the NanoString nCounter^®^ SPRINT system. We transfected A549 cells to express mCherry-fused DDX3X, mCherry-fused DDX3X(qmNESα), or mCherry alone, then sorted the mCherry-expressing populations and assayed mRNA transcript levels 24 h post-hPIV-3 or mock infection. Transcript levels were monitored using the PanCancer Immune Profiling RNA probe library. After internal normalization and discarding low-count data, we measured transcription across a total of 730 human genes relevant to immunity and cancer (Table S1). The vast majority of genes were downregulated in uninfected cells expressing mCherry-DDX3X compared to mCherry alone (Figure 6A), suggesting DDX3X may act as a ‘brake’ on immune genes at steady-state. Consistent with this idea, many of the genes were upregulated in uninfected cells expressing mCherry-DDX3X(qmNESα), implying that DDX3X’s ability to traffic between the nucleus and cytoplasm, dependent on its exportin-1 recognized N-terminal NESα, is central to this function. As expected, viral infection resulted in strong activation of many of these genes in cells expressing wild-type, but not in cells expressing nuclear export defective, DDX3X. This is reflected in the distant clustering of the wild-type DDX3X samples between steady-state and infection, as opposed to the much closer clustering of the DDX3X(qmNESα) samples in the absence or presence of infection (Figure 6A. See also principal component analysis in Figure 6B). The data show that the anti-hPIV-3 inflammatory response in lung tissue is overwhelmingly characterized by the induction of IFN-β and ISGs including proinflammatory cytokines and chemoattractants for neutrophils (e.g., *CXCL1*, *CXCL2*, *CXCL3*, *IL1A, IL6*, *IL8, PTGS2,* and *SAA1*) and T-cells (e.g., *CCL5*, *CCL20*, *CXCL10*, *CXCL11*, *IL6*, and *IL8*), as well as innate immune signaling proteins (e.g., *MX1, IFI27, IFIT1, IFIT2, IRF7, ISG15, ISG20, STAT1,* and *TLR8*) and inducers of apoptosis (e.g., *IL1B, IFI27*, and *IFIT2*) (Figure 6C). 

Comparison of the mRNA levels for cells ectopically expressing DDX3X with or without a functional NES revealed clear differences in the subsets of ISGs expressed. In resting cells, ectopic expression of DDX3X(qmNESα) resulted in increased mRNA levels of 523 genes (Figure 6D). Only 173 of these showed similar effects upon overexpression of wild-type DDX3X. There were an additional set of 49 genes, distinct from those impacted by DDX3X(qmNESα), showing elevated levels upon overexpression of wild-type DDX3X (Figure 6D). Upon hPIV-3 infection, wild-type DDX3X-expressing cells distinctly upregulated 192 genes, whereas only 85 were distinctly upregulated in DDX3X(qmNESα)-expressing cells (Figure 6D). Overall, these data highlight that nucleocytoplasmic trafficking of DDX3X is critically important in regulating gene induction during viral infection, with elevated nuclear expression of DDX3X impacting the resting state transcriptome as well as that in response to viral infection.

### 3.10. Nuclear DDX3X Contributes to IFNB1 Transcription and Influences ISG Subset Induction

Chromatin-immunoprecipitation experiments indicate DDX3X can associate with the *IFNB1* promoter [10]. Our observation that expression of nuclear-localizing DDX3X(qmNESα) led to elevated levels of IFN-β secretion in response to hPIV-3 infection compared to wild-type DDX3X (Figure 5B) correlated nicely with the fact that a large number (217) of the genes upregulated upon overexpression of DDX3X(qmNESα) were ISGs, including *IFNB1* itself. For the latter, hPIV-3-infected A549 cells expressing either DDX3X or DDX3X(qmNESα) showed enhanced *IFNB1* transcription versus mCherry alone (normalized induction of 0.924 and 0.942 versus 0.870, respectively), with DDX3X(qmNESα) showing the greatest overall induction (Table S1). Consistent with the idea that DDX3X’s nucleocytosolic distribution modulates its role as a brake on immune induction at rest, uninfected cells overexpressing DDX3X showed lower *IFNB1* transcription than cells overexpressing mCherry only, whereas cells overexpressing DDX3X(qmNESα) once again showed enhanced *IFNB1* transcription (normalized induction of –0.979, –0.909, and –0.847, respectively) (Table S1). The nuclear trafficking of DDX3X thus appears to modulate *IFNB1* gene transcription, modulated by exportin-1 binding to the DDX3X N-terminal NESα in a Ran-GTP-dependent manner.

Results for DDX3X- and DDX3X(qmNESα)-overexpressing cells were compared directly to gain insight into specific effects on ISGs during hPIV-3 infection (Figure 6E). Strikingly, wild-type DDX3X-overexpressing cells showed higher expression of genes encoding proinflammatory factors upregulated during hPIV-3 infection (e.g., *CCL5, CXCL1, CXCL2, CXCL3, CXCL10, CXCL11, IL1A, IL1B, IL6, IL8,* and *SAA1*) compared to DDX3X(qmNESα)-expressing cells (Figure 6E and Table S1). Genes encoding other cytokines and receptors associated with inflammation (e.g., *CCL3*, *CCL3L1, CCL4, CCL17,* and *CCR5*), apoptosis (e.g., *TP53* and *CASP3*), and autophagy (e.g., *LAMP3*) showed similar results. In contrast, DDX3X(qmNESα)-expressing cells showed higher mRNA levels for *IFNB1* as well as effector genes specifically related to IFN-I expression (e.g., *IKBKE*/IKKε, *RIPK2, TBK1*) and antigen presentation to T lymphocytes (e.g., *HLA-DMB*, *CD244, ICOS*) (Figure 6E and Table S1). This supports the idea that nuclear export-inhibited DDX3X can help drive transcription contributing to IFN-I-mediated immunity and T-cell recruitment/activation, whereas wild-type nucleocytoplasmic trafficking DDX3X supports a broader-ranging, and thereby more effective, array of antiviral responses, assisting in coordinating the innate and adaptive immune responses to infection.

To further validate the above results, we examined protein expression levels of a subset of the above genes in addition to *IFNB1*, representing a broad range of cellular pathways and antiviral defenses. Changes in transcriptional activity of *CASP3*, *RIPK2*, *LAMP2,* and *TBK1* (Figure 6E) were reflected in corresponding changes in expression of encoded proteins as determined by immunoblot (Figure 6F), giving confidence that our overall dataset for IFN-1/ISG induction/expression is robust. 

## 4. Discussion

DDX3X is a key host cellular factor in the RLR signaling cascade and is implicated in the replication strategy of a large and growing list of evolutionarily divergent pathogens of significance to human health, including hepatitis B virus [37], hepatitis C virus [38], influenza A virus [39], Japanese encephalitis virus [40], West Nile virus [41], dengue virus [42], and HIV-1 [17]. Understanding the link between DDX3X subcellular localization and the host- and pathogen-directed roles of DDX3X are central to unlocking novel strategies to target DDX3X activity in infection.

Consistent with a NES-dependent interaction, we and others [8,17,18,19,20] have shown that DDX3X’s nuclear export is inhibited by the exportin-1-specific inhibitor LMB, which blocks cargo protein binding and trafficking by covalently modifying the NES-binding interface of exportin-1 [15,16]. Correspondingly, our qCLSM, co-immunoprecipitation, and analytical ultracentrifugation sedimentation velocity data confirms DDX3X harboring a nonfunctional NES is incapable of binding exportin-1 even at supraphysiological concentrations, and reciprocally, Ran-GTP is strictly required for DDX3X binding to exportin-1. Previously, DDX3X’s nuclear export was attributed to a unique exportin-1-dependent mechanism requiring DDX3X helicase domain residues 260–517, but neither a recognized exportin-1 NES nor the Ran-GTP gradient [17]. However, consistent with Brennan et al. [20] our results do not support this finding. Collectively, we confirm the mechanism of DDX3X’s exportin-1-dependent nuclear export is typical of other receptor–cargo interactions and aligns with that of An3, the *Xenopus laevis* orthologue of DDX3X, which shares 87% sequence identity overall and an identical NES within the N-terminus of DDX3X [43]. Notably, the key hydrophobic residues of the DDX3X/An3 NES are also conserved down to the *Saccharomyces cerevisiae* orthologue Ded1p, which also undergoes exportin-1-mediated nuclear export in a NES- and Ran-GTP-dependent manner [44].

Although exportin-1 is an exporter of DDX3X we observe residual DDX3X in the cytosol following inactivation of the NES or LMB treatment. One explanation may be that DDX3X utilizes other nuclear export pathways in addition to the exportin-1 pathway. The C-terminal region of DDX3X was previously reported to mediate binding and nuclear export by NXF1 [18]. We did not observe any contribution of the DDX3X C-terminal region to its nucleocytosolic distribution in this study, but cannot formally exclude the possibility that other nuclear export receptors may bind and traffic DDX3X in certain circumstances. We propose it is equally plausible that the nuclear import of DDX3X is weak at steady-state, but is then enhanced during specific events or stages of the cell cycle [20]. Importantly, the nuclear import mechanism of DDX3X remains unknown and warrants further study. Previous studies [20], as well as our own, have only implicated regions involved in the nuclear import of DDX3X.

Our study suggests invasive RNA is a trigger for authentic nuclear accumulation of DDX3X, which supports IFN-β induction and secretion. Nearly all proinflammatory genes strongly activated during hPIV-3 infection were positively associated with expression of wild-type DDX3X, while IFN-β itself and a particular subset of IFN-I signaling and effector genes were more strongly expressed when DDX3X accumulated more strongly in the nucleus. This reveals that regulated trafficking of DDX3X between the nucleus and cytosol is crucial for controlling IFN-β levels, at least in response to hPIV-3 infection, as well as supporting transcription of a particular subset of IFN-I signaling and effector genes in order to amplify the IFN-I response. 

While we do not exclude the possibility that endogenous DDX3X expression levels may play a role, we propose the following model of DDX3X trafficking-dependent immune regulation based on our observations. In the resting state, DDX3X acts as a ‘brake’ on immune gene induction to prevent unnecessary immune activation. However, upon exposure to invasive RNA during acute-phase virus infection, DDX3X supports cytosolic signaling events leading to IFN-I expression, redistributing to the nucleus to help drive transcription contributing to IFN-I mediated immunity and T-cell recruitment/activation. Notably, the nuclear export of DDX3X via exportin-1 is critical for maximal gene induction, and thereby presumably results in a more effective innate and adaptive immune response to infection, and as demonstrated in our hPIV-3 infectious model. 

Our results indicate that DDX3X plays a hitherto unrecognized antiviral role in hPIV-3 replication that is contingent upon its export into the cytosol, and seemingly independent of its role in IFN-β induction. Consistent with this finding, IFN-α and type III IFN (IL29A, IL-28A and/or IL28B), as opposed to IFN-β, are reported to have anti-hPIV-3 action [45,46], and type III-IFN receptor deficiencies increase susceptibility to hPIV-3 infection [47]. Despite nuclear export-deficient DDX3X being permissive to hPIV-3 replication, LMB treatment, which blocks the nuclear export of all exportin-1 cargos, including DDX3X, suppressed hPIV-3 replication. This suggests that the nuclear export of unknown host and/or hPIV-3 viral proteins plays a pivotal role in hPIV-3 replication, and that compounds such as LMB specifically targeting exportin-1 in this context may be effective against hPIV-3, as reported for other viruses [23,36,48].

DDX3X subcellular localization is central to its function in antiviral immunity and hence paramount to the infectivity of microbes that exploit DDX3X as an essential host cofactor. For example, HIV-1 Rev requires nuclear DDX3X to export HIV-1 transcripts to the cytoplasm [17], whilst cytoplasmic DDX3X is required for RSV M2 translation [49]. This suggests that host-orientated agents that alter the nuclear import/export of DDX3X are likely effective antiviral agents. Indeed, inhibitors of exportin-1, such as those developed by Karyopharm^®^ Therapeutics [50], that inhibit nuclear export of all cargoes recognized by exportin-1 bearing a NES can be efficacious broad-spectrum antivirals (e.g., against RSV and influenza infection). Accordingly, LMB treatment inhibited hPIV-3 replication in the current study, and Hendra virus [23], RSV [35], and Venezuelan equine encephalitis virus [36] in previous studies, suggesting their replication requires exportin-1-dependent nuclear export. However, there are currently no cargo-specific nuclear export inhibitors, which are critically important in reducing the cytotoxic effects of global inhibition of exportin-1. We anticipate our work exploring the exportin-1 mediated nuclear export of DDX3X and understanding its functional relevance in directing antiviral immune signaling outcomes will support the pursuit of DDX3X-specific nuclear export inhibitors that will have implications for viruses of significance to human health such as HIV-1 and RSV.

## Figures and Tables

**Table 1:** Hydrodynamic properties of recombinant DDX3X(1–580) or mutants thereof, exportin-1, Ran-GTP, and their resulting complexes. Related to Figure 3.

**Individual Proteins**	***M*_r_^1^**	***M*^2^**	***s*_20,w_^3^**	***f/f*_0_^4^**
Exportin-1	123386	117954	4.9	1.7
Ran-GTP	24423	25682	2.2	1.3
DDX3X(1-168)	19403	15856	1.7	1.7
DDX3X(1-580)	65945	71566	3.6	1.7
DDX3X(1-580)(qmNESα)	65743	70076	3.7	1.6
**Protein Complexes**	***M*_r_^1^**	***M*^2^**	***s*_20,w_^3^**	***f/f*_0_^4^**
Exportin-1-Ran-GTP	147809	133003	5.1	1.7
DDX3X(1-168) + Exportin-1-Ran-GTP	167212	128675	7.8	1.9
DDX3X(1-580) + Exportin-1-Ran-GTP	213754	179465	7.0	1.6

^1^ Relative molecular weight (*M_r_*) calculated from the amino acid sequences. ^2^ Molar mass (*M*) determined from the ordinate maximum of *c*(*M*) distribution best fits (data not shown). ^3^ Standardized sedimentation coefficient from the ordinate maximum of the *c*(*s*) distribution best fits (Figure 3). ^4^ Frictional coefficient calculated from *s*_20,w_ using the ⊽ method employing SEDNTERP.

## Supplementary Materials

The following are available online at https://www.mdpi.com/2073-4409/8/10/1181/s1, Table S1: Complete normalized, z-transformed NanoString analysis dataset.

## References

[1] Valiente-Echeverria F., Hermoso M.A., Soto-Rifo R., Valiente-Echeverría F., Soto-Rifo R. (2015). RNA helicase DDX3: At the crossroad of viral replication and antiviral immunity. Rev. Med. Virol..

[2] Solis M., Nakhaei P., Jalalirad M., Lacoste J., Douville R., Arguello M., Zhao T., Laughrea M., Wainberg M.A., Hiscott J. (2011). RIG-I-mediated antiviral signaling is inhibited in HIV-1 infection by a protease-mediated sequestration of RIG-I. J. Virol..

[3] Sabbah A., Bose S. (2009). Retinoic acid inducible gene I Activates innate antiviral response against human parainfluenza virus type 3. Virol. J..

[4] Cortez K.J., Erdman D.D., Peret T.C.T., Gill V.J., Childs R., Barrett A.J., Bennett J.E. (2001). Outbreak of human parainfluenza virus 3 infections in a hematopoietic stem cell transplant population. J. Infect. Dis..

[5] Sydnor E.R., Greer A., Budd A.P., Pehar M., Munshaw S., Neofytos D., Perl T.M., Valsamakis A. (2012). An outbreak of human parainfluenza virus 3 infection in an outpatient hematopoietic stem cell transplantation clinic. Am. J. Infect. Control..

[6] Gu L., Fullam A., McCormack N., Hohn Y., Schroder M. (2017). DDX3 directly regulates TRAF3 ubiquitination and acts as a scaffold to co-ordinate assembly of signalling complexes downstream from MAVS. Biochem. J.

[7] Oshiumi H., Sakai K., Matsumoto M., Seya T. (2010). DEAD/H BOX 3 (DDX3) helicase binds the RIG-I adaptor IPS-1 to up-regulate IFN-beta-inducing potential. Eur. J. Immunol..

[8] Schröder M., Baran M., Bowie A.G. (2008). Viral targeting of DEAD box protein 3 reveals its role in TBK1/IKKepsilon-mediated IRF activation. EMBO J..

[9] Heaton S.M., Borg N.A., Dixit V.M. (2016). Ubiquitin in the activation and attenuation of innate antiviral immunity. J. Exp. Med..

[10] Soulat D., Bürckstümmer T., Westermayer S., Goncalves A., Bauch A., Stefanovic A., Hantschel O., Bennett K.L., Decker T., Superti-Furga G. (2008). The DEAD-box helicase DDX3X is a critical component of the TANK-binding kinase 1-dependent innate immune response. EMBO J..

[11] Mohr D., Frey S., Fischer T., Güttler T., Görlich D. (2009). Characterisation of the passive permeability barrier of nuclear pore complexes. EMBO J..

[12] Fornerod M., Ohno M., Yoshida M., Mattaj I.W. (1997). CRM1 is an export receptor for leucine-rich nuclear export signals. Cell.

[13] Fukuda M., Asano S., Nakamura T., Adachi M., Yoshida M., Yanagida M., Nishida E. (1997). CRM1 is responsible for intracellular transport mediated by the nuclear export signal. Nature.

[14] Engelsma D., Bernad R., Calafat J., Fornerod M. (2004). Supraphysiological nuclear export signals bind CRM1 independently of RanGTP and arrest at Nup358. EMBO J..

[15] Sun Q., Carrasco Y.P., Hu Y., Guo X., Mirzaei H., Macmillan J., Chook Y.M. (2013). Nuclear export inhibition through covalent conjugation and hydrolysis of Leptomycin B by CRM1. Proc. Natl. Acad. Sci. USA.

[16] Kudo N., Matsumori N., Taoka H., Fujiwara D., Schreiner E.P., Wolff B., Yoshida M., Horinouchi S. (1999). Leptomycin B inactivates CRM1/exportin 1 by covalent modification at a cysteine residue in the central conserved region. Proc. Natl. Acad. Sci. USA.

[17] Yedavalli V.S., Neuveut C., Chi Y.H., Kleiman L., Jeang K.T. (2004). Requirement of DDX3 DEAD Box RNA helicase for HIV-1 Rev-RRE export function. Cell.

[18] Lai M.C., Lee Y.H.W., Tarn W.Y. (2008). The DEAD-box RNA helicase DDX3 associates with export messenger ribonucleoproteins as well as tip-associated protein and participates in translational control. Mol. Biol. Cell.

[19] Sekiguchi T., Iida H., Fukumura J., Nishimoto T. (2004). Human DDX3Y, the Y-encoded isoform of RNA helicase DDX3, rescues a hamster temperature-sensitive ET24 mutant cell line with a DDX3X mutation. Exp. Cell Res..

[20] Brennan R., Haap-Hoff A., Gu L., Gautier V., Long A., Schröder M. (2018). Investigating nucleo-cytoplasmic shuttling of the human DEAD-box helicase DDX3. Eur. J. Cell Biol..

[21] Sharma D., Bhattacharya J. (2010). Evolutionary constraints acting on DDX3X protein potentially interferes with Rev-Mediated nuclear export of HIV-1 RNA. PLoS ONE.

[22] Mahboobi S.H., Javanpour A.A., Mofrad M.R.K. (2015). The interaction of RNA helicase DDX3 with HIV-1 Rev-CRM1-RanGTP complex during the HIV replication cycle. PLoS ONE.

[23] Atkinson S.C., Audsley M.D., Lieu K.G., Marsh G.A., Thomas D.R., Heaton S.M., Paxman J.J., Wagstaff K.M., Buckle A.M., Moseley G.W. (2018). Recognition by host nuclear transport proteins drives disorder-to-order transition in Hendra virus V. Sci. Rep..

[24] Rusinova I., Forster S., Yu S., Kannan A., Masse M., Cumming H., Chapman R., Hertzog P.J. (2013). Interferome v2.0: An updated database of annotated interferon-regulated genes. Nucl. Acids Res..

[25] Fraser J.E., Rawlinson S.M., Heaton S.M., Jans D.A. (2016). Dynamic nucleolar targeting of Dengue virus polymerase NS5 in response to extracellular pH. J. Virol..

[26] Laue T.M., Shah B.D., Ridgeway T.M., Pelletier S.L., Harding S.E. (1992). Computer-aided interpretation of analytical sedimentation data for proteins. Analytical Ultracentrifugation in Biochemistry and Polymer Science.

[27] Schuck P. (2000). Size-distribution analysis of macromolecules by sedimentation velocity ultracentrifugation and Lamm equation modeling. Biophys. J..

[28] Moseley G.W., Roth D.M., DeJesus M.A., Leyton D.L., Filmer R.P., Pouton C.W., Jans D.A. (2007). Dynein light chain association sequences can facilitate nuclear protein import. Mol. Biol. Cell.

[29] Li J.-Z., Chen X., Gong X.-L., Hu H.-Y., Shi D., Lu Y.-M., Qiu L., Lu F., Hu Z.-L., Zhang J.-P. (2013). Identification of a functional nuclear localization signal mediating nuclear import of the zinc finger transcription factor ZNF24. PLoS ONE.

[30] Van De Garde M.D.B., Pas S.D., Van Oord G.W., Gama L., Choi Y., De Man R.A., Boonstra A., Vanwolleghem T. (2017). Interferon-alpha treatment rapidly clears Hepatitis E virus infection in humanized mice. Sci. Rep..

[31] Askjaer P. (1998). The specificity of the CRM1-Rev nuclear export signal interaction is mediated by RanGTP. J. Biol. Chem..

[32] Hübner S., Xiao C.-Y., Jans D.A. (1997). The protein kinase CK2 site (Ser111/112) enhances recognition of the simian virus 40 large T-antigen nuclear localization sequence by importin. J. Biol. Chem..

[33] Huang T.T., Kudo N., Yoshida M., Miyamoto S. (2000). A nuclear export signal in the N-terminal regulatory domain of Ikappa Balpha controls cytoplasmic localization of inactive NF-kappa B/Ikappa Balpha complexes. Proc. Natl. Acad. Sci. USA.

[34] Senchi K., Matsunaga S., Hasegawa H., Kimura H., Ryo A. (2013). Development of oligomannose-coated liposome-based nasal vaccine against human parainfluenza virus type 3. Front. Microbiol..

[35] Ghildyal R., Ho A., Dias M., Soegiyono L., Bardin P.G., Tran K.C., Teng M.N., Jans D.A. (2009). The respiratory syncytial virus matrix protein possesses a Crm1-mediated nuclear export mechanism. J. Virol..

[36] Lundberg L., Pinkham C., Baer A., Amaya M., Narayanan A., Wagstaff K.M., Jans D.A., Kehn-Hall K. (2013). Nuclear import and export inhibitors alter capsid protein distribution in mammalian cells and reduce Venezuelan Equine Encephalitis Virus replication. Antiviral. Res..

[37] Yu S., Chen J., Wu M., Chen H., Kato N., Yuan Z. (2010). Hepatitis B virus polymerase inhibits RIG-I- and Toll-like receptor 3-mediated beta interferon induction in human hepatocytes through interference with interferon regulatory factor 3 activation and dampening of the interaction between TBK1/IKKepsilon and DDX3. J. Gen. Virol..

[38] Ariumi Y., Kuroki M., Abe K., Dansako H., Ikeda M., Wakita T., Kato N. (2007). DDX3 DEAD-Box RNA helicase is required for hepatitis C virus RNA replication. J. Virol..

[39] Raman S.N.T., Liu G., Pyo H.M., Cui Y.C., Xu F., Ayalew L.E., Tikoo S.K., Zhou Y. (2016). DDX3 interacts with influenza A virus NS1 and NP proteins and exerts antiviral function through regulation of stress granule formation. J. Virol..

[40] Li C., Ge L.L., Li P.P., Wang Y., Dai J.J., Sun M.X., Huang L., Shen Z.Q., Hu X.C., Ishag H. (2014). Cellular DDX3 regulates Japanese encephalitis virus replication by interacting with viral un-translated regions. Virology.

[41] Chahar H.S., Chen S., Manjunath N. (2013). P-body components LSM1, GW182, DDX3, DDX6 and XRN1 are recruited to WNV replication sites and positively regulate viral replication. Virology.

[42] Khadka S., Vangeloff A.D., Zhang C., Siddavatam P., Heaton N.S., Wang L., Sengupta R., Sahasrabudhe S., Randall G., Gribskov M. (2011). A physical interaction network of dengue virus and human proteins. Mol. Cell. Proteom..

[43] Askjaer P., Bachi A., Wilm M., Bischoff F.R., Weeks D.L., Ogniewski V., Ohno M., Niehrs C., Kjems J., Mattaj I.W. (1999). RanGTP-regulated interactions of CRM1 with nucleoporins and a shuttling DEAD-Box helicase. Mol. Cell. Biol..

[44] Hauk G., Bowman G.D. (2015). Formation of a trimeric Xpo1-Ran[GTP]-Ded1 exportin complex modulates ATPase and helicase activities of Ded1. PLoS ONE.

[45] Eberle K.C., McGill J.L., Reinhardt T.A., Sacco R.E. (2015). Parainfluenza virus 3 blocks antiviral mediators downstream of the interferon lambda receptor by modulating Stat1 phosphorylation. J. Virol..

[46] Zhao H., De B.P., Das T., Banerjee A.K. (1996). Inhibition of human parainfluenza virus-3 replication by interferon and human MxA. Virology.

[47] E Dorman S., Uzel G., Roesler J., Bradley J.S., Bastian J., Billman G., King S., Filie A., Schermerhorn J., Holland S.M. (1999). Viral infections in interferon-gamma receptor deficiency. J. Pediatr..

[48] Perwitasari O., Johnson S., Yan X., Register E., Crabtree J., Gabbard J., Howerth E., Shacham S., Carlson R., Tamir S. (2016). Antiviral efficacy of verdinexor in vivo in two animal models of influenza A virus infection. PLoS ONE.

[49] Easton A., Gould P., Marsh A. (2012). Use of DDX3X inhibitors for the treatment of pneumovirus infections. US Patent.

[50] Pickens J.A., Tripp R.A. (2018). Verdinexor Targeting of CRM1 is a promising therapeutic approach against RSV and influenza viruses. Viruses.

